# Strategy to Enhance Anticancer Activity and Induced Immunogenic Cell Death of Antimicrobial Peptides by Using Non-Nature Amino Acid Substitutions

**DOI:** 10.3390/biomedicines10051097

**Published:** 2022-05-09

**Authors:** Yu-Huan Cheah, Chun-Yu Liu, Bak-Sau Yip, Chih-Lung Wu, Kuang-Li Peng, Jya-Wei Cheng

**Affiliations:** 1 Department of Medical Science, Institute of Biotechnology, National Tsing Hua University, Hsinchu 300, Taiwan; s107080710@m107.nthu.edu.tw (Y.-H.C.); s109080536@m109.nthu.edu.tw (C.-Y.L.); g15004@hch.gov.tw (B.-S.Y.); s103080578@m103.nthu.edu.tw (C.-L.W.); richard850210@gapp.nthu.edu.tw (K.-L.P.); 2 Department of Neurology, National Taiwan University Hospital Hsinchu Branch, Hsinchu 300, Taiwan

**Keywords:** antimicrobial peptides, oncolytic peptides, cancer, membrane integrity, bulky non-nature amino acid, DAMPs

## Abstract

There is an urgent and imminent need to develop new agents to fight against cancer. In addition to the antimicrobial and anti-inflammatory activities, many antimicrobial peptides can bind to and lyse cancer cells. P-113, a 12-amino acid clinically active histatin-rich peptide, was found to possess anti-*Candida* activities but showed poor anticancer activity. Herein, anticancer activities and induced immunogenic cancer cell death of phenylalanine-(Phe-P-113), β-naphthylalanine-(Nal-P-113), β-diphenylalanine-(Dip-P-113), and β-(4,4′-biphenyl)alanine-(Bip-P-113) substituted P-113 were studied. Among these peptides, Nal-P-113 demonstrated the best anticancer activity and caused cancer cells to release potent danger-associated molecular patterns (DAMPs), such as reactive oxygen species (ROS), cytochrome c, ATP, and high-mobility group box 1 (HMGB1). These results could help in developing antimicrobial peptides with better anticancer activity and induced immunogenic cell death in therapeutic applications.

## 1. Introduction

Antimicrobial peptides (AMPs) are found in the host innate defense mechanisms and play important roles in combating microbial infections [1,2,3,4]. AMPs may work alone or in combination with antibiotics to diminish drug-resistant pathogens and improve the therapeutic effects of antibiotics [1,5,6,7,8,9,10]. Recent progress of antibiotic AMP conjugates also displays exceptional in vitro and in vivo efficacies [11,12]. Most AMPs possess two critical characteristics: a net cationicity to interact with negatively charged microbial surfaces and an amphipathic structure to incorporate into microbial cell membranes. Other than broad-spectrum antimicrobial activities, many AMPs also exert lipopolysaccharide (LPS) neutralization, as well as anticancer activities [13,14,15].

The histidine-rich peptide P-113 (AKRHHGYKRKFH-NH_2_) was derived from saliva protein histatin 5 [16]. P-113 has demonstrated its efficacy in a clinical trial for HIV patients with oral candidiasis [17]. However, P-113 lost its activity in high salt conditions [18]. The antimicrobial activities of P-113 under high-salt conditions can be restored by replacing the histidine amino acids with the bulky non-natural amino acids: β-naphthylalanine (Nal), diphenylalanine (Dip), and (4,4′-biphenyl)alanine (Bip) [18,19]. In addition, these P-113 derivatives all displayed enhanced serum proteolytic stability, in vitro and in vivo LPS neutralization, and synergistic activities with antibiotics [9,18,19].

Cancer has been found to be the leading cause of death in many countries [9]. The insurgence of chemotherapeutic resistance is still a major cause of treatment failure of cancer patients [14]. Hence, the development of novel therapeutics with high specificity toward cancer cells and/or treatments stimulating the patients’ own immune system to fight against cancer cells is the utmost concern in the cancer research field. Similar to the disruption of the negatively charged microbial cell membranes, some AMPs can bind to the negatively charged phosphatidylserine moieties exposed on the outer surface of cancer cell plasma membranes and cause the lysis of cancer cells [15]. In addition to direct lysis of cancer cells, the membrane-disruptive or membrane-lytic properties of AMPs may help conventional standard-of-care chemotherapeutics to fight against drug-resistant cancer cells [20]. Moreover, the release of tumor antigens and potent danger-associated molecular patterns (DAMPs) were found after treatment with these AMPs [15,21]. The release of danger signals (DAMPs) may induce complete regression and long-term protective immune responses against cancer, as previously reported [22]. However, the mode of action of the interactions between AMPs and cancer cells or the rules governing the design of AMPs with anticancer activities are still not clear.

Based on our previous studies of the interactions between microbial pathogens and P-113 and its derivatives [9,18,19], we suspected that the substitution of bulky non-natural amino acids may also affect the anticancer activity of P-113. Herein, we determined the cytotoxic effect of P-113 and its bulky non-natural amino acids substituted derivatives against five cancer cell lines. In addition, we measured the release of reactive oxygen species (ROS), high-mobility group box 1 (HMGB1), cytochrome c, and ATP after the treatment of cancer cells with P-113 derivatives.

## 2. Materials and Methods

### 2.1. Materials

All peptides were purchased from Kelowna International Scientific Inc. (Taipei, Taiwan). The identity of the peptides was checked by electrospray mass spectroscopy, and the purity (>95%) was assessed by high-performance liquid chromatography (HPLC). Sequences of P-113 and its derivatives are shown below:

P-113 (Ac-AKR **His His** GYKRKF **His**-NH_2_)

Phe-P-113 (Ac-AKR **Phe Phe** GYKRKF **Phe**-NH_2_)

Nal-P-113 (Ac-AKR **Nal Nal** GYKRKF **Nal**-NH_2_)

Bip-P-113 (Ac-AKR **Bip Bip** GYKRKF **Bip**-NH_2_)

Dip-P-113 (Ac-AKR **Dip Dip** GYKRKF **Dip**-NH_2_)

Dulbecco’s modified Eagle’s medium (DMEM), Roswell Park Memorial Institute 1640 (RPMI 1640) medium, and Trypsin-EDTA were purchased from Gibco-Life Technologies (New York, NY, USA). Fetal bovine serum (FBS) was purchased from Biological industries (Beit HaEmek, Israel). Dimethyl sulfoxide (DMSO), MTT (3-(4,5-dimethylthiazol-2-yl)2.5-diphenyl tetrazolium bromide), L-Glutamine, penicillin, streptomycin, propidium iodide, and Hoechst 33342 were purchased from Sigma-Aldrich (St. Louis, MO, USA). JC-1 (5,5′,6,6′-Tetrachloro-1,1′,3,3′-tetraethyl-imidacarbocyanine iodide) was purchased from Dojindo Molecular Technologies (Rockville, MD, USA). CCCP (Carbonyl cyanide 3-chloro-phenylhydrazone) was purchased from Cayman Chemical Company (Ann Arbor, MI, USA).

### 2.2. Cell Lines and Cultural Conditions

The three non-small cell lung cancer cell lines, H1975, PC 9, A549, and oral squamous cell carcinoma cell line OECM-1 were cultured in RPMI 1640 medium supplemented with 10% fetal bovine serum, 2 mM L-glutamine and 1% penicillin/ streptomycin. The oral cancer cell line C9 was cultured in DMEM with 10% fetal bovine serum, 2 mM L-glutamine, and 1% penicillin/ streptomycin. All the cell lines were cultured in the humidified incubator containing 5% CO_2_ at 37 °C.

### 2.3. Cell Viability Assay

The MTT assay was applied for cell viability test according to previous protocol [23]. The cells were seeded in the 96-well plate at 5000 cells/well under the environment of humidified 5% CO_2_ and the temperature of 37 °C for 24 h. The medium was removed from the well before the fresh medium containing different concentrations of peptides was added and incubated for 24 h. Medium treated with PBS served as control group. All peptides were diluted serially from 100 µM to 0.78 µM in ddH_2_O before adding in the medium. After 24 h of peptide treatment, fresh medium with 10% MTT solution (5 mg/mL) was incubated for 4 h. After medium was removed, 100 µL DMSO was added to dissolve the formazan crystal. Absorbance was measured at 570 nm using a microplate reader (TECAN Sunrise ELISA Reader). The cell survival was calculated from the treated cells relative to the control (100% viable cells) using the mean of three independent experiments and expressed as a 50% inhibitory concentration (IC_50_).

The percentage of cell viability was calculated using the following formula:

(1) $$\mathrm{Cell}\;\mathrm{viability}\;\%=\frac{\mathrm{Absorbance}\;\mathrm{of}\;\mathrm{sample}\;-\;\mathrm{Blank}}{\mathrm{Absorbance}\;\mathrm{of}\;\mathrm{control}\;-\;\mathrm{Blank}}\;\times \;100\%$$

### 2.4. Kinetic Analysis

A total of 5000 cells were incubated with Nal-P-113, Bip-P-113, and Dip-P-113 at 2× IC_50_ for 0.5, 1, 2, 3, and 4 h. After treatment, the MTT solution was further incubated for 3 h. Absorbance was measured at 570 nm on the microplate reader (TECAN Sunrise ELISA Reader) after the dissolution of the formazan crystal in 100 µL DMSO solution. The cell survival rate was calculated as aforementioned for cell viability assay.

### 2.5. Propidium Iodide (PI)/Hoechst 33342 Staining

To examine the membranolytic activities of peptides, the PI/Hoechst 33342 staining was performed in this study. The PI dye was used to stain damaged/dead cells, and Hoechst 33342 was used to specifically stain the nuclei of living cells [21,24]. PC 9 cells were seeded at 20,000 cells/well in a RPMI medium and allowed to adhere for 48 h. Then, the cells were replaced with serum-free RPMI medium and then treated with 2× IC_50_ peptides at 37 °C for 120 min. Then, the PI and Hoechst 33342 were added at a final concentration of 1 μg/mL for 20 min. PC 9 cells were observed using the inverted fluorescent microscope Zeiss equipped with a 40× oil objective lens (Carl Zeiss, Jena, Germany). The experiments were repeated three times independently.

### 2.6. Fluorescence Microscopy of JC-1 Staining

PC 9 cells were seeded at 40,000 cells/well in a RPMI medium and allowed to adhere for 24 h. Then, the cells were replaced with serum-free RPMI medium and treated with 2× IC_50_ or 150 µM CCCP (Carbonyl cyanide 3-chloro-phenylhydrazone) at 37 °C for 120 min. The JC-1 dye was added at a final concentration of 1 μg/mL for 45 min. PC 9 cells were observed using the inverted fluorescent microscope Zeiss equipped with a 20× oil objective lens (Carl Zeiss, Jena, Germany). The experiments were repeated three times independently.

### 2.7. Measurement of Extracellular ATP

PC 9 cells were seeded at the density of 10,000 cells/ well in the 96-well plate and allowed to adhere for 48 h. Cells were treated with Nal-P-113, Bip-P-113, and Dip-P-113 at 2× IC_50_ for 0.5, 1, 1.5, and 2 h. Untreated cells were served as the control group. After being treated for different time points (0.5, 1, 1.5, and 2 h), the supernatant of cells was analyzed using the ENLITEN ATP luciferase assay kit (Promega, Madison, WI, USA). The level of extracellular ATP chemiluminescence was measured using the Wallac VICTOR 3 Multilabel plate reader (PerkinElmer, Shelton, CT, USA).

### 2.8. Measurement of Cellular ROS

The 2′,7′-dichlorofluorescin diacetate (DCFDA) Cellular ROS Detection Assay Kit (Abcam, Cambridge, UK, ab113851) was used to analyze the ROS release in the PC 9 cell line. DCFDA is a cell-permeable non-fluorescent probe, which diffuses into the cytoplasm and then forms a non-fluorescent moiety through deacetylation by cellular esterase. The non-fluorescent moiety could be oxidized by cellular ROS to form the fluorescent product DCF [21]. PC 9 cells were seeded at the density of 25,000 cells/ well in the 96-well black plate and allowed to adhere for 24 h. Cells were washed with a 100 μL/well of PBS to remove the RPMI 1640 medium and incubated with 100 µL of the 25 µM DCFDA solution for 45 min at 37 °C in the dark condition. An amount of 100 µL/ well of PBS buffer was washed again for the removal of 25 µM DCFDA solution. The PC 9 cells were treated with Nal-P-113, Bip-P-113, and Dip-P-113 at 2× IC_50_ for 2 h. The untreated cells were used as a control. The fluorescence intensity of cellular ROS was measured using the Wallac VICTOR 3 Multilabel plate reader (PerkinElmer, Shelton, CT, USA) at an excitation wavelength of 485 nm and an emission wavelength of 535 nm.

### 2.9. Detection of Mitochondrial Cytochrome C Release to Cytosol

PC 9 cells were seeded with 10,000 cells/well in the 96-well plate and allowed to adhere for 48 h and then treated with Nal-P-113, Bip-P-113, and Dip-P-113 at 2× IC_50_ for 0.5, 1, 1.5, 2, and 4 h. Untreated cells were used as a control group. Supernatants were collected after centrifugation at 16,000× *g* for 10 min. Samples were analyzed using the Quantikine ELISA Rat/ Mouse Cytochrome c Immunoassay (R & D Systems, Minneapolis, MN, USA) according to the manufacturer’s protocol. The optical density at the wavelength of 450 nm of each well was determined using the microplate reader (TECAN Sunrise ELISA Reader).

### 2.10. Detection of Extracellular HMGB1

The PC 9 cells were seeded at the density of 25,000 cells/well and left to adhere for 48 h. Cells were treated with Nal-P-113, Bip-P-113, and Dip-P-113 at 2× IC_50_ for 1, 2, and 4 h. After treatment of the peptides, the supernatant and lysate of the cells were collected separately for further experiments. The supernatant was collected after centrifugation at the speed of 1400× *g*. The cell lysate was washed twice using PBS before the sonication of the pellet using a bio-disruptor, and the disrupted pellet was centrifuged again for 10 min under 14,000 rpm at 4 °C. The concentration of the samples was quantified using the Bradford assay. Both supernatants and cell lysate were loaded and run on the sodium dodecyl sulfate polyacrylamide gel electrophoresis (SDS-PAGE) and were then electro transferred to a polyvinylidene difluoride (PVDF) membrane (Merck-Millipore, Burlington, MA, USA) in an electro-blot system, 30 V, 400 mA, for 75 min. The membrane was incubated in blocking buffer (5% skim milk, TBST buffer) for 1 h at room temperature and washed in TBST buffer three times. The anti-HMGB1 antibody (Abcam, Cambridge, UK, ab18256) was incubated overnight at 4 °C in the dilution ratio of 1:1000. The secondary antibody of a rabbit (diluted in 1:10,000) was incubated following rinsing with PBST three times for 2 h the next day at room temperature before being developed using a luminol reagent and imaged by a detected system (ImageQuant LAS 4000 mini).

### 2.11. Statistical Analysis

All the statistical data represent the average of three independent experiments with standard deviation (mean ± SD) and each experiment set consisting of three replications. The evaluation of statistical analyses was calculated using one-way ANOVA and Student’s *t*-tests using GraphPad Prism version 8.0 (San Diego, CA, USA). Significant differences were represented with thresholds of * *p* < 0.05; ** *p* < 0.01; *** *p* < 0.001.

## 3. Results

### 3.1. Anticancer Activities of P-113 and Its Derivatives

**Figure 1:**
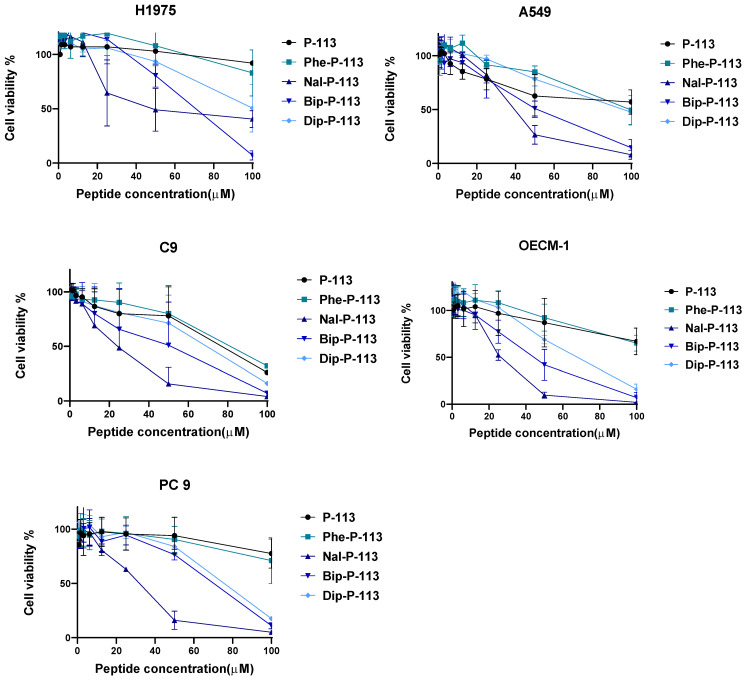
Anticancer activities of P-113, Phe-P-113, Nal-P-113, Bip-P-113, and Dip-P-113 against various cancer cell lines by MTT cell viability assay. Data represent mean ± SD of three independent experiments.

Five cancer cell lines, including three non-small cell lung cancer cell lines (H1975, A549, PC 9) and two oral cancer cell lines (C9, OECM-1), were used to test the cytotoxic effects of P-113 and its derivatives. H1975 is the non-small cell lung carcinoma cells, A549 is the adenocarcinomic alveolar basal epithelial cells, and PC 9 is the non-small cell lung cancer with EGFR mutation. C9 and OECM-1 cell lines are oral squamous cell carcinoma and gingival epidermoid carcinoma, respectively. P-113 and Phe-P-113 peptides have the highest survival rates (>60%) among every cell line tested, even when the peptide concentration had reached 100 µM (Figure 1). Nal-P-113, Bip-P-113, and Dip-P-113 peptides exhibited significant anticancer activities against these five cell lines (Figure 1). IC_50_ values of P-113 and its derivatives are shown in Table 1. As can be seen from Table 1, the order of the anticancer activities is Nal-P-113 > Bip-P-113 > Dip-P-113 > Phe-P-113 > P-113.

The toxicity of the peptides was determined previously by measuring cell death using MTT assays against human fibroblasts (HFW) [19]. The results indicated that even at 100 μg/mL, Nal-P-113, Bip-P-113, and Dip-P-113 only caused less than 10% cell death.

### 3.2. Cancer Cell Killing Kinetic Analysis 

**Figure 2:**
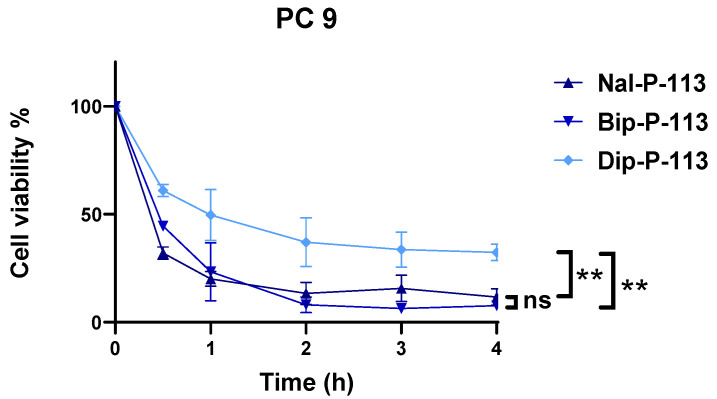
Time killing analysis of Nal-P-113, Bip-P-113, and Dip-P-113 against PC 9 cell line. Data represent mean ± SD of three independent experiments. ** *p* < 0.01 (Nal-P-113 vs. Dip-P-113; Bip-P-113 vs. Dip-P-113), ns = no significant differences (Nal-P-113 vs. Bip-P-113).

The evaluation of the time killing kinetic of Nal-P-113, Bip-P-113, and Dip-P-113, which displayed excellent anticancer activity against PC 9 cell line, was employed by MTT cell viability assay. Two-fold half inhibitory concentration (2× IC_50_) was chosen for three independent experiments, and the results were recorded from 30 min to 4 h. As shown in Figure 2, Nal-P-113 and Bip-P-113 started to kill cancer cells after 30 min of peptide incubation and reduced the cell survival rate to below 50%. Within 2 h, Nal-P-113 and Bip-P-113 had effectively killed more than 80% of the cells, which makes both of them the most effective peptides for anticancer activity. On the other hand, cancer cells treated with Dip-P-113 had the highest cellular survival rate at around 60% in 30 min. The overall cell survival rate of the group treated with Dip-P-113 was the highest compared to Nal-P-113 and Bip-P-113.

### 3.3. Loss of Plasma Membrane Integrity

**Figure 3:**
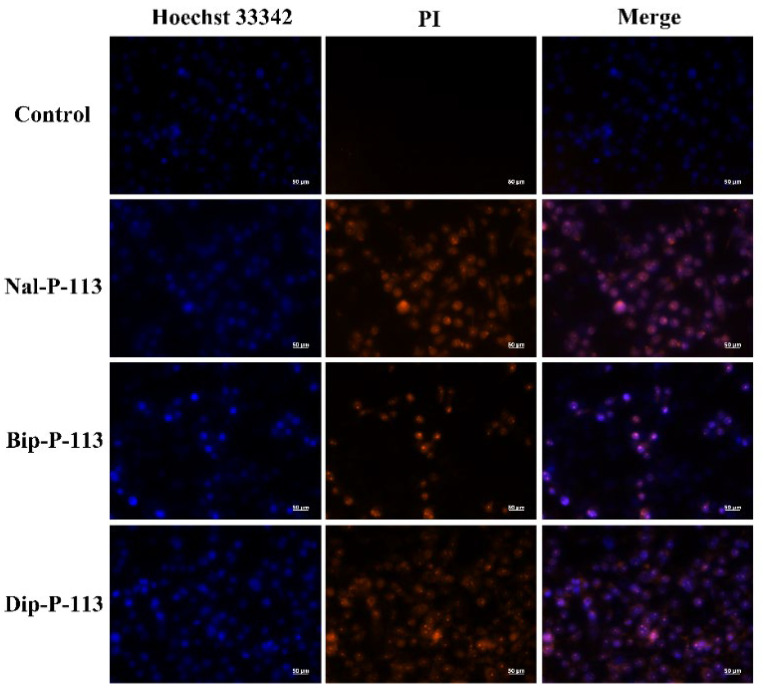
Fluorescence images of PC 9 cells treated with or without Nal-P-113, Bip-P-113, and Dip-P-113 at 2× IC_50_ for 120 min and stained with PI (red) and nuclear dye Hoechst 33342 (blue) using fluorescence microscopy. Scale bar represents 50 μm. The experiments were repeated three times independently.

The DNA binding fluorescent probe PI can only enter necrotic cells with a damaged plasma membrane [21]. In this study, PI was used to demonstrate that Nal-P-113, Bip-P-113, and Dip-P-113 can cause damage to the plasma membrane integrity of PC 9 cells and increase the amount of PI entering into PC 9 cells (Figure 3).

### 3.4. Mitochondrial Membrane Interactions

**Figure 4:**
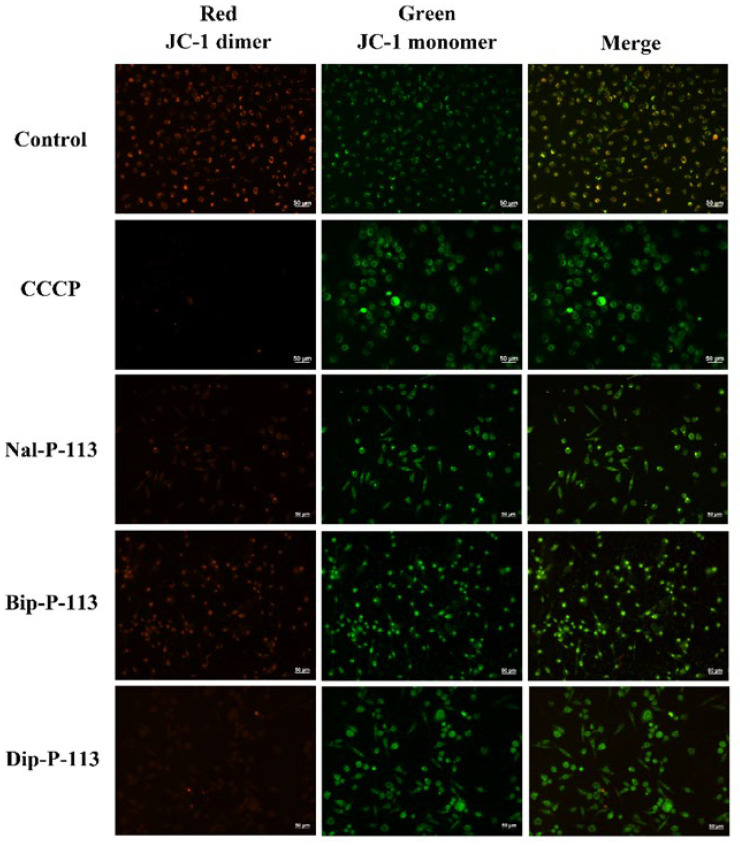
Fluorescence images of PC 9 cells treated with 2× IC_50_ Nal-P-113, Bip-P-113, and Dip-P-113 for 120 min and stained with JC-1. Samples treated with CCCP served as a positive control. Scale bar represents 50 μm. The experiments were repeated three times independently.

JC-1 is a cationic dye and is known to be localized exclusively in mitochondria [25]. Under sufficient mitochondrial membrane potential, JC-1 will form J-aggregates with a specific red fluorescence emission. Losing mitochondrial membrane potential (dysfunction) due to interactions with membrane-lytic peptides will lead to a decrease in JC-1 accumulation. JC-1 will then form J-monomers with a typical green fluorescence. Figure 4 shows Nal-P-113, Bip-P-113, and Dip-P-113 induced changes of the mitochondrial membrane potential in PC 9 cells, indicating that these peptides interact with the mitochondria membrane and cause dysfunction of mitochondria.

### 3.5. Production of Reactive Oxygen Species (ROS) 

**Figure 5:**
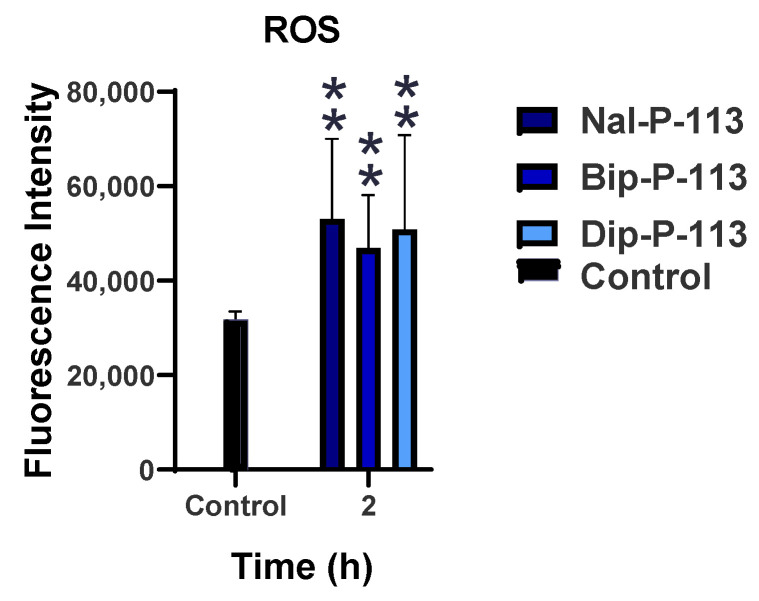
ROS release after the treatment with 2× IC_50_ of Nal-P-113, Bip-P-113, and Dip-P-113 detected by DCFDA cellular ROS detection kit. The untreated cells were used as control. Results are presented as mean ± SD of three independent experiments, ** *p* < 0.01 compared with control.

Reactive oxygen species (ROS) is an important indicator in immunogenic cell death, and the level of ROS production may be caused by dysfunctional mitochondria. Figure 5 demonstrates that PC 9 cells treated with Nal-P-113, Bip-P-113, and Dip-P-113 had increasing fluorescence intensities to the level of more than 40,000. These results indicated that Nal-P-113, Bip-P-113, and Dip-P-113 may cause the damage of mitochondria and promote ROS production.

**Figure 6:**
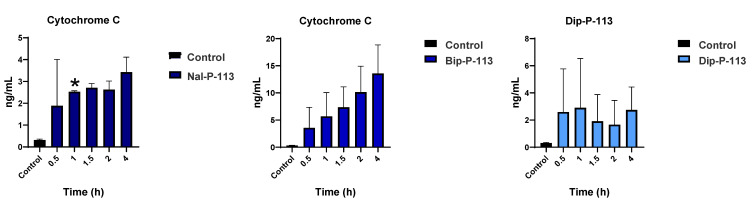
The cytochrome c secretion was quantified using ELISA assay. Results are presented as mean ± SD of three independent experiments. The cells were treated with Nal-P-113, Bip-P-113, and Dip-P-113 peptides at 2× IC_50_ concentration for 0.5, 1, 1.5, 2, and 4 h, respectively. Untreated cells were used as a control. * *p* < 0.05 compared with control.

Detection of Mitochondrial Cytochrome C release into Cytosol. The cytochrome c release was a part of the mitochondrial danger-associated molecular patterns. Therefore, the amount of cytochrome c released into the supernatant was measured to investigate the capability of Nal-P-113, Bip-P-113, and Dip-P-113 to induce the mitochondrial DAMPs. Five time points (0.5, 1, 1.5, 2, and 4 h) were used in the ELISA assay to quantify the amount of cytochrome c released, and the results were shown in Figure 6. Bip-P-113 induced a relatively high amount of cytochrome c compared to Nal-P-113 and Dip-P-113. The cytochrome c release for the Bip-P-113-treated group was more than double the amount of Nal-P-113- and Dip-P-113-treated groups. It is observed that the trend of cytochrome c release in Bip-P-113- and Nal-P-113-treated PC 9 cells was escalating from 30 min to 4 hours’ time course, in contrast to the Dip-P-113 treatment group, which had a fluctuating trend. In the Bip-P-113-treated group, the amount of cytochrome c release came to a maximal value of nearly 15 ng/mL at 4 h, while the minimum value of 3 ng/mL at 30 min was observed. The highest amounts of cytochrome c detected in the supernatant for Nal-P-113 and Dip-P-113 were around 3.5 ng/mL and lower than 2 ng/mL for the least. These results suggested that these three peptides have the capability to cause cancer cells to release cytochrome c into the supernatant and thus triggering the features of immunogenic cell death.

### 3.6. Extracellular ATP Release

**Figure 7:**
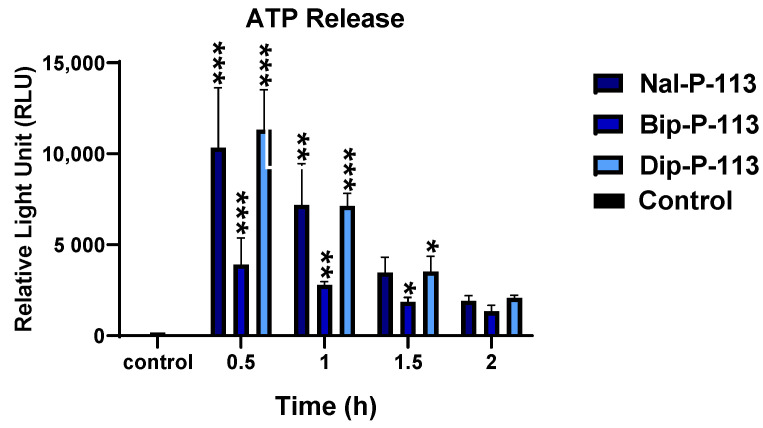
ATP release of PC 9 cells after treatment with Nal-P-113, Bip-P-113, and Dip-P-113. Untreated cells were served as the control group. Results are presented as mean ± SD of three independent experiments, * *p* < 0.05; ** *p* < 0.01; *** *p* < 0.001 compared with control.

ATP release was recognized as the iconic feature for immunogenic cell death. Following treatment with 2× IC_50_ of Nal-P-113, Bip-P-113, and Dip-P-113 for 0.5, 1, 1.5, and 2 h, ATP release from PC 9 cells was measured using the luciferin-luciferase assay (Figure 7). The relative light unit (RLU) recorded the highest value for all three peptides at the treatment time of 0.5 h and declining over time. Although all three peptides reached the peak at 0.5-h, Bip-P-113 had the lowest ATP release, while Nal-P-113 and Dip-P-113 displayed similar amounts of ATP release. PC 9 cells treated with Nal-P-113 and Dip-P-113 showed greatest ATP release in the supernatant after incubating for 0.5 h, and the ATP amount detected in the supernatant decreased gradually over time.

### 3.7. Extracellular HMGB1 Release

**Figure 8:**
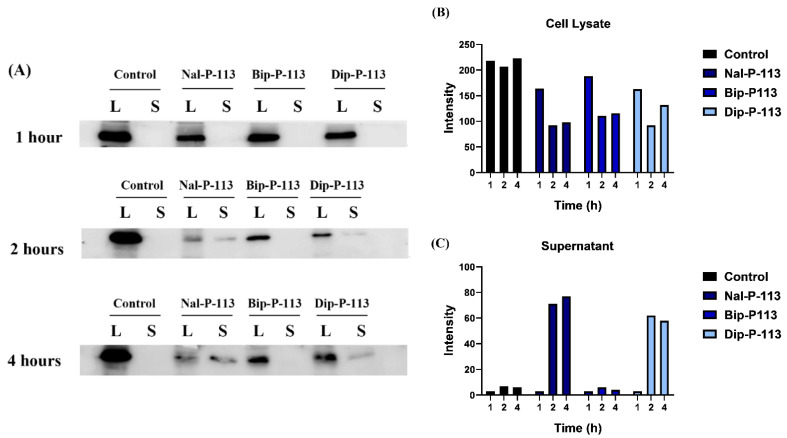
HMGB1 protein secreted from cell lysate to supernatant after incubation with Nal-P-113, Bip-P-113, and Dip-P-113. (**A**) The Western blot images of the PC 9 cells were treated with 2× IC_50_ Nal-P-113, Bip-P-113, and Dip-P-113 peptides for 1, 2, and 4 h, respectively. Untreated cells were served as a control group. L and S represented cell lysate and supernatant, respectively. The relative band intensities of HMGB1 for each group from cell lysate (**B**) and supernatant (**C**).

The protein band of HMGB1 (29 kDa) for both cell lysate and supernatant was used to detect HMGB1 protein release after treatment with Nal-P-113, Bip-P-113, and Dip-P-113. No HMGB1 protein was observed in the supernatant of PC 9 cells in the 1 h treatment group (Figure 8). HMGB1 protein can clearly be seen in the supernatant of the Nal-P-113 and Dip-P-113 treatment group after 2 and 4 h (Figure 8). On the other hand, HMGB1 protein can hardly be detected in the supernatant of the Bip-P-113 treatment group after 2 and 4 h. Nevertheless, after 2 h, the cell lysate band of the Nal-P-113, Bip-P-113, and Dip-P-113 treatment groups all started to fade.

## 4. Discussion

AMPs have been proposed as promising antimicrobial and antitumor agents owing to their unique mechanism of action [14]. AMPs kill bacterial and cancer cells through the disruption or lysis of cell membranes instead of acting on a specific target, such as DNA or enzyme. Previously, we have shown that Nal-P-113, Bip-P-113, and Dip-P-113 possessed enhanced salt resistance, serum proteolytic stability, LPS neutralization, as well as synergism with antibiotics against drug-resistant bacteria [7,14,15,22]. Nal-P-113, Bip-P-113, and Dip-P-113, unlike P-113, were also found to target *C. albicans’* cell surface instead of translocating into cells [26]. In the present study, compared to the anticancer abilities of Nal-P-113, Bip-P-113, and Dip-P-113, only P-113 and Phe-P-113 demonstrated weak anticancer activities against the five cell lines studied. On the other hand, Nal-P-113, Bip-P-113, and Dip-P-113 were found to target the cell membrane of cancer cells and cause damage to plasma membrane integrity. Among these non-nature amino acids, Bip is the longest and Dip is the widest, with Nal having an intermediate length and width relative to Bip and Dip [27]. Structure–activity relationships of P-113, Phe-P-113, Nal-P-113, Dip-P-113, and Bip-P113 indicated that Bip-P-113 displayed superior antimicrobial and LPS neutralizing activities compared to Nal-P-113 and Dip-P-113 [28]. The lengthy sidechain of Bip may create extra hydrophobic interactions with the lipid A motif of LPS. However, for the five cancer cell lines studied, Nal-P-113 appeared to have the best anticancer activity and kill cancer cells rapidly. It is possible that the intermediate size and shape of Nal creates suitable hydrophobic interactions with the membrane of cancer cells.

**Figure 9:**
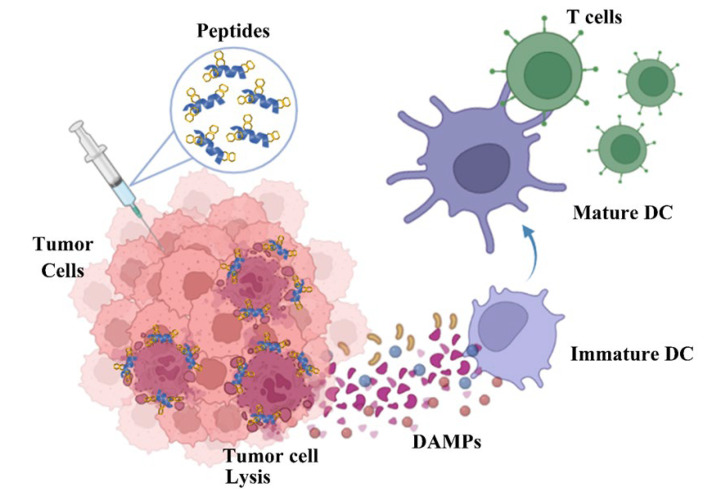
Proposed mechanism of anticancer action for the designed peptides. The intratumoral administration of Nal-P-113 (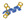) induced tumor cellular lysis through membrane destabilization, caused the release of danger-associated molecular patterns (DAMPs), including ATP (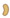), HMGB1 (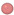), and ROS (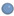), as well as tumor antigens into the tumor microenvironment. Thereafter, these DAMPs recruited the immature dendritic cells (DCs) and further induced the maturation of dendritic cells. The mature DCs were then priming for antigen presentation to T cells and subsequent antitumor immune responses.

Immunogenic cell death is the type of cell death that can induce an immune response. Cell death can be classified into two major groups: programed/regulated cell death (such as apoptosis, autophagy, and necroptosis) and accidental cell death due to non-physiological reasons (necrosis) [29]. Apoptosis normally causes cell shrinkage, plasma membrane blebbing, and formation of apoptotic bodies. Necrosis, on the other hand, causes increase in cell volume, loss of plasma membrane integrity, and leakage of cellular contents [29]. Radiotherapy and some chemotherapeutic agents can induce immunogenic cell death of cancer cells [30]. After the occurrence of immunogenic cell death of cancer cells, danger-associated molecular patterns (DAMPs), such as HSP70, ATP secretion, cytochrome c release, and HMGB1 protein, could be induced [31]. The host immune system against cancer cells can be activated by sensing the presence of DAMPs by dendritic cells [32]. Additionally, the immunogenic cell death response’s efficacy is regulated by parameters such as how efficient the dendritic cells phagocytize dead cancer cells, as well as how efficiently the cytotoxic T cells target cancer cells by tumor antigen-specific killing (Figure 9). For example, PKHB1, a thrombospondin-1 peptide mimic, can induce DAMPs in breast cancer cell lines, and the supernatant of PKHB1-killed breast cancer cells can induce maturation of bone-marrow-derived DCs and elicit antitumor T cell responses [33]. Some AMPs, such as LTX-315 [21], ΔM4 [34], and TP4 [35], were shown to induce a rapid plasma membrane disruption of cancer cells and cause the release of DAMPs into the cell supernatant. In an immunocompetent mouse model, the local injection of LTX-315 into tumors resulted in complete regression and specific anticancer immune responses with long-term protective immunity [36,37]. Moreover, the results of a recent clinical trial demonstrated that LTX-315 has an acceptable safety profile and is clinically active via inducing necrosis and CD8+ T-cell infiltration into the solid tumor microenvironment [38]. An abscopal effect was also found following treatment with LTX-315 [38].

In addition to anticancer activities, Nal-P-113, Bip-P-113, and Dip-P-113 also possess enhanced ability to induce immunogenic cell death. The present studies suggested that Nal-P-113 and Dip-P-113 might cause cancer cells to undergo the necrosis pathway, and HMGB1 protein can be detected as early as 2 h post-treatment. On the other hand, Bip-P-113 might depend on apoptotic cell death, as HMGB1 protein was not detected in the Western blot analysis. Undetectable HMGB1 protein in the Bip-P-113-treated group might be due to inactivation of HMGB1 protein by oxidation, and further studies should be carried out for a more concrete argument.

Reactive oxygen species (ROS) production is believed to be an important component in the intracellular danger signaling to govern immunogenic cell death [39]. ROS overproduction may help open the mitochondrial permeability transition pore and cause the leakage of mitochondrial components into the cytosol and outside of the cell. These molecules may then act as DAMPs to trigger the immune response. For example, as ROS rise, molecules such as cytochrome c may be released from mitochondria and activate the caspase cascade, which then triggers apoptosis. Excessive ROS promotes necrotic cell death. The present results also supported that Nal-P-113 and Dip-P-113 might have undergone the necrotic cell death pathway. The hypothesis that Bip-P-113 caused the cancer cells to die through the apoptotic pathway is further supported by evidence of a steady decrease in cytosolic ATP associated with a large number of dead cells [40]. ATP depletion may cause the switching from apoptotic cell death to necrotic cell death [40].

High levels of cytochrome c release were found with Bip-P-113. The mitochondria releases cytochrome c when it is damaged, and the cytochrome c then contributes to apoptotic cell death. Both Nal-P-113 and Dip-P-113 did not cause much cytochrome c release, and this might be due to the rapid switching form of cell death, from apoptosis to necrosis cell death.

In conclusion, our results demonstrated that P-113 derivatives with bulky non-nature amino acid substitutions display compelling anticancer activities against five cancer cell lines, including three non-small cell lung cancer cell lines (H1975, A549, PC 9) and two oral cancer cell lines (C9, OECM-1). Moreover, these peptides caused necrosis of cancer cells and triggered the damaged cancer cells to release potent danger-associated molecular patterns (DAMPs), such as reactive oxygen species (ROS), cytochrome c, ATP, and HMGB1. The release of DAMPs may lead to the maturation and activation of dendritic cells of the adaptive immune system (Figure 9). Among these peptides, Nal-P-113 demonstrated the best anticancer activities and induced immunogenic cell death. Our results should be useful in the development of new antimicrobial peptides and peptidomimetics with enhanced anticancer activities for potential therapeutic applications.

## Figures and Tables

**Table 1:** IC_50_ (μM) of P-113 and its derivatives.

	Peptide	P-113	Phe-P-113	Nal-P-113	Bip-P-113	Dip-P-113
Cell Line						
H1975		110.0	106.6	58.32	63.65	100.4
A549		106.6	99.06	38.22	48.43	96.43
C9		84.27	80.49	21.37	41.97	67.89
OECM-1		140.6	119.9	25.91	42.47	62.94
PC 9		>200	172.7	28.11	64.19	71.45

## Data Availability

Not applicable.

## Abbreviations

AMPs	antimicrobial peptides
Phe	phenylalanine
Nal	β-naphthylalanine
Dip	β-diphenylalanine
Bip	β-(4,4′-biphenyl)alanine
DAMPs	danger-associated molecular patterns
ROS	reactive oxygen species
ATP	adenosine triphosphate
HMGB1	high-mobility group box 1
HPLC	high-performance liquid chromatography
FBS	fetal bovine serum
DMSO	dimethyl sulfoxide
MTT	3-(4,5-dimethylthiazol-2-yl)2.5-diphenyl tetrazolium bromide
JC-1	5,5′,6,6′-Tetrachloro-1,1′,3,3′-tetraethyl-imidacarbocyanine iodide
CCCP	carbonyl cyanide 3-chloro-phenylhydrazone
ELISA	enzyme-linked immunosorbent assay
PI	propidium iodide
